# Supplementary material for: Association of RNA-modification “writer” genes with prognosis and response to immunotherapy in patients with low-grade glioma
Source: PLoS One. 2023 Jan 17;18(1):e0279119. doi: 10.1371/journal.pone.0279119 (PMC9844866; doi:10.1371/journal.pone.0279119)
Supplement: S1 File — (DOCX) [file pone.0279119.s001.docx]

***Supplemental Material***

**1 Supplementary Methods**

**Harris Hawks Optimizer**

The HHO algorithm is a novel metaheuristic algorithm developed by simulating the cooperative foraging behavior of Harris Hawks. Intelligent foraging is realized through tracking, encircling, and attacking. The specific description of each stage is as follows:

**Exploration phase**

In the global exploration stage, Harris Hawks roost on large trees or telegraph poles and capture prey by waiting, searching, and attacking. The mathematical expression is given as follows:

$x_{i}^{t+1}=\left\{\begin{matrix} x_{rand}^{t}-r_{1}*|x_{rand}^{t}-2*r_{2}*x_{i}^{t}|,q\geq0.5 \\ (x_{rabbit}-x_{mean}^{t})-r_{3}*(lb+r_{4}*(ub-lb)),\text{ }q<0.5 \end{matrix}\right.$ (1)

where $x_{rand}^{t}$ is a random Harris Hawk in the t-generation population, $x_{i}^{t}$ is the i^th^ Harris Hawk in the t-generation population, and $x_{rabbit}$ is the Harris Hawk in the optimal position (as prey) in the current population. $q$ is a conversion factor whose value is a random number in the range of [0,1], $r_{1}$, $r_{2}$, $r_{3}$, and $r_{4}$ are random numbers in the range of [0, 1], and $ub$ and $lb$ are the upper and lower bounds of the population, respectively. $x_{mean}^{t}$ is the mean value of the position in the current population, and the calculation method is as follows:

$x_{mean}^{t}=\frac{1}{n}\sum_{i=1}^{n} x_{i}^{t}$ (2)

where $n$ is the population size.

**Transition from exploration to** **exploitation**

In the HHO, the transition from global exploration to local exploitation is controlled by the energy control factor *E* (escaping energy). The transition method is shown in Eq. (3):

$E=2*E_{0}*(1-t/T)$ (3)

where $E_{0}$ is a random number in the range of [-1, 1], and $t$ and $T$ are the current and maximum iterations, respectively.

**Exploitation stage**

In the exploitation stage, each individual Harris Hawk randomly chooses one of the following escape strategies based on the energy (energy factor) of the prey when it escapes.

(i) *Soft* *besiegement.* When $r\geq0.5$ and $|E|\geq0.5$, the prey has enough energy but no chance to escape the encirclement; in this case, Harris Hawks employ a soft besiegement approach to hunt the prey. The former part can be mathematically modeled as:

$x_{i}^{t+1}=\Delta x_{i}^{t}-E*|J*x_{rabbit}-x_{i}^{t}|$ (4)

$\Delta x_{i}^{t}=x_{rabbit}-x_{i}^{t}$ (5)

where $\Delta x_{i}^{t}$ is the vector distance between the optimal individual (prey) in the population and the current Harris Hawk individual, $r_{5}$ is a random number satisfying the uniform distribution of [0,1], and $J=2*(1-r_{5})$ is the jumping length when the prey escapes.

(ii) *Hard besiege.* When $r\geq0.5$ and $|E|<0.5$, the prey does not have enough energy nor the chance to escape the encirclement; this is when the Harris Hawks employ a hard besiegement approach to hunt the prey.

$x_{i}^{t+1}=x_{rabbit}-E*|\Delta x_{i}^{t}|$ (6)

(iii) *Soft besiegement with progressive rapid dives.* When $r<0.5$ and $|E|\geq0.5$, the prey has enough energy and chance to escape the encirclement; in this case, the Harris Hawks search for the prey in two steps. The first step is executed using Eq. (7). Second, if the position of the Harris Hawk is not improved after the first step is performed, a second step update mode is implemented. The mathematical expression for the first step strategy is as follows:

$y=x_{rabbit}-E*|J*x_{rabbit}-x_{i}^{t}|$ (7)

The second step update method is expressed as follows:

$z=y+s*levy(\dim)$ (8)

where $\dim$ is the dimension, $s$ is the random vector with the dimension $\dim$, and $levy$ is a random number satisfying a Lévy distribution. Its definition is shown in Eq. (9).

$levy(x)=0.01\times\frac{u\times\sigma}{|\mu|^{\frac{1}{\beta}}},\sigma=(\frac{\Gamma(1+\beta)\times\sin(\frac{\pi\beta}{2})}{\Gamma(\frac{1+\beta}{2})\times\beta\times2^{(\frac{\beta-1}{2})}})^{\frac{1}{\beta}}$ (9)

where $u$ and $\mu$ are random numbers satisfying [0,1], and $\beta$ is a constant value (1.5).

For an optimization problem that solves the minimum value, the mathematical model is given as follows:

$x_{i}^{t+1}=\left\{\begin{matrix} y,\text{ if }f\text{(y)<}f\text{(}x_{i}^{t}\text{) } \\ z,\text{ if }f\text{(z)<}f\text{(}x_{i}^{t}\text{) } \end{matrix}\right.$ (10)

where $f$ is the fitness value for solving the optimization problem.

(iv) *Hard besiege with progressive rapid dives.* When $r<0.5$ and $|E|<0.5$, the prey has a chance to escape the encirclement but has insufficient energy. At this time, the Harris Hawks form a hard encirclement around the prey before attacking, and then implement the attack strategy. For an optimization problem that solves the minimum value, the mathematical model is given as follows:

$x_{i}^{t+1}=\left\{\begin{matrix} y,\text{ if }f\text{(y)<}f\text{(}x_{i}^{t}\text{) } \\ z,\text{ if }f\text{(z)<}f\text{(}x_{i}^{t}\text{) } \end{matrix}\right.$ (11)

where,

$y=x_{rabbit}-E*|J*x_{rabbit}-x_{mean}^{t}|$ (12)

$z=y+s*levy(\dim)$ (13)

# VNLHHO for feature selection of gene expression data

## Coding rules

The basic HHO is only suitable for solving continuous optimization problems; the selection of features from gene expression data involves binary discrete data. To apply the HHO to binary coding and solve practical problems, the most commonly used activation function (i.e., binary discretization) in existing literature is the Sigmoid function. This activation function works well only when the feature difference is not particularly large. Where there is a significant difference between the positive and negative directions, a greater shift from the previous position is required. To overcome this shortcoming, we use Eqs. (14) and (15) to perform binary discretization on continuous data.

$s(v_{i,j})=\left(1-e^{-A(i)/(t\cdot T)}\right)+\left|\frac{e^{v_{i,j}}-e^{-v_{i,j}}}{e^{v_{i,j}}+e^{-v_{i,j}}}\right|$ (14)

$x_{i,j}=\left\{\begin{matrix} 1,s(v_{i,j})>rand(0,1) \\ 0,else \end{matrix}\right.$ (15)

where $A(i)$ is the statistical value of the number of iterations required by individual *i* to obtain a better optimal solution. When the individual $x_{i}$ finds a better optimal solution than the current one, $A(i)$=0; otherwise, $A(i)\text{=}A(i)\text{+1}$. T and t are the maximum and current numbers of iterations, respectively.

## Primary selection of gene features based on F-score

More than 90% of the genes in gene expression data are irrelevant to tumor classification. Therefore, it is necessary to remove these genes without reducing the classification accuracy. The F-score algorithm has advantages such as fast computation and simple implementation. While maintaining a high classification accuracy, this algorithm can select effective features and information from high-dimensional gene expression data and is often used for primary selection of high-dimensional data. The primary selection process for tumor gene expression data in terms of the F-score is as follows:

For an original dataset $data=\{(x_{1},y_{1}),(x_{2},y_{2}),...,(x_{n},y_{n})\}$, where $x_{i}=(x_{i,1},x_{i,2},...x_{i,\dim})$ is the dim-dimensional feature vector, $y_{i}\in\text{（0,1,...,m）}$, m is the classification label, and n is the number of samples, the calculation formula between each feature and label category in the sample is shown in Eq. (16):

$Fscore_{i}=\frac{\sum_{k=1}^{m} (\bar{x}_{i}^{k}-\bar{x}_{i})^{2}}{\sum_{k=1}^{m} \left[\frac{1}{p_{i}^{k}-1}\sum_{j=1}^{p_{i}^{k}} (x_{i,j}^{k}-\bar{x}_{_{i}}^{k})^{2}\right]},i=1,2,...,n$ (16)

where $p_{i}^{k}$ is the number of i-th feature of the k-th sample, $x_{i,j}^{k}$ is the j-th training sample of the i-th feature of the k-th sample, and $\bar{x}_{i}$ and $\bar{x}_{_{i}}^{k}$ represent the mean of all the samples on the i-th feature and the mean of the k-th sample on the i-th feature.

## Fitness function

Tumor gene feature selection is a type of combinatorial optimization problem, which belongs to an NP problem. The intelligent optimization algorithm is the most cost-effective method for obtaining the approximate optimal solution to such problems. To solve such problems with the intelligent optimization algorithm, it is necessary to set a reasonable fitness function as the evaluation standard. However, tumor gene feature selection is a multi-objective optimization problem, which requires considering both the classification accuracy and the number of features. Therefore, for a good trade-off between the minimum number of features and the maximum classification accuracy when setting the fitness function, the objective function is calculated using Eqs. (17) and (18).

$\text{Acc=}\frac{num_{c}}{num_{c}+num_{e}}\times100\%$ (17)

$\text{fitness=}\alpha\text{*Acc+(1-}\alpha\text{)*(1-}\frac{I}{M}\text{)}$ (18)

where $\text{Acc}$ denotes the percentage of correctly classified examples. The numbers of correctly and incorrectly classified samples are indicated by ($num_{c}$) and ($num_{e}$) respectively. Further, $I$ is the number of selected features, $M$ is the total number of features, and $\alpha$ is the weight coefficient (0.99 in this study).

## Update mode of VNLHHO for gene feature selection

In the VNLHHO algorithm, individuals can keep moving in the continuous search space given the existence of position vectors with real continuous neighborhoods. To enable Harris Hawk individuals to move in the binary search space, it is necessary to improve the speed update mode of the VNLHHO algorithm. Therefore, the updated data values are converted into binary values using Eqs. (14) and (15) to adapt to the tumor gene feature selection process. In the VNLHHO, the update mode mainly includes three stages:

***Learning from*** ***the position outside the neighborhood:*** Harris Hawk individuals acquire knowledge by learning the average ability (position) of individuals outside the neighborhood. At this stage, its speed is calculated using Eq. (19):

$v_{i}^{t+1}=rand()\cdot(x_{i}^{t}-avg_{i}^{t})$ (19)

where $avg_{i}$ is the information of individuals with an average position outside the neighborhood.

***Learning from the position inside the neighborhood*:** Harris Hawk individuals acquire knowledge by learning the abilities of optimal individuals inside the neighborhood. At this stage, its speed is calculated using Eq. (20):

$v_{i}^{t+1}=rand()\cdot(x_{i}^{t}-best_{k}^{t})$ (20)

where $best_{k}^{t}$ is the position of the optimal individual inside the neighborhood.

***Speed update based on the basic HHO algorithm:*** In the basic HHO algorithm, the updating of the individual positions of the Harris Hawks is based on a combination of the original position and the updating speed of the position. In the VNLHHO, the updating speed of the position is selected in this study.

## Mutation operators of VNLHHO

If the effectiveness of the search fails to improve after multiple updates ($A(i)>\lim$, where $\lim$ is a limited number of iterations, this algorithm takes 1/4T), we perform a mutation operation on the individuals to prevent the algorithm from falling into a local optimum. The mutation mode is shown in Eq. (21):

$x_{i,j}^{'}\text{=}\left\{\begin{matrix} x_{i,j}\text{ or }x_{best,j},\text{succ=1} \\ (x_{i,j}\text{ }x\text{or }x_{best,j})\text{ or (}x_{i,j}\text{ or }x_{k,j}\text{) }k\neq i,else \end{matrix}\right.$ (21)

where succ is a marking function of whether the individual position is improved. If the position is improved, then succ=0; otherwise, succ=1.

## Gene feature selection process of VNLHHO

Figure S9 shows the gene feature selection process of the VNLHHO based on the above description, and the implementation steps are as follows:

Step 1: Initialize the population and parameters. Specifically, m Harris Hawks initial populations with n-dimensional space are generated randomly, and each Harris Hawks individual is simulated as a binary string. Suppose that any Harris Hawk i can be represented by a discrete string containing “0” and “1” at any moment (iteration t) as $x_{i}^{t}=(x_{i}^{1,t},x_{i}^{2,t},...,x_{i}^{n,t}),i=1,2,...,m;t=1,2,...,T$, where “1” in the string means that the corresponding feature is selected and “0” means that the feature is not selected.

Step 2: Evaluate the fitness value. Calculate the fitness value of each individual according to Eq. (18), and identify the optimal individual $x_{best}^{t}$ in the population.

Step 3: Execute the learning strategy from the position outside the neighborhood. Convert the continuous solution generated by Eq. (19) into a binary solution. Evaluate the fitness value and identify the optimal individual $x_{best}^{t}$ in the population.

Step 4: Execute the learning strategy from the position inside the neighborhood. Evaluate the fitness value and identify the optimal individual $x_{best}^{t}$ in the population.

Step 5: Execute the speed update strategy based on the basic HHO algorithm. Evaluate the fitness value and identify the optimal individual $x_{best}^{t}$ in the population.

Step 6: If the mutation operator condition is met, execute the mutation operators using Eq. (21). Evaluate the fitness value and identify the optimal individual $x_{best}^{t}$ in the population.

Step 7: Continue the iteration process. If it fails to meet the limit on the number of iterations set by the initialization, return to step 3, or output $x_{best}$.

**2 Supplementary Figures**

**
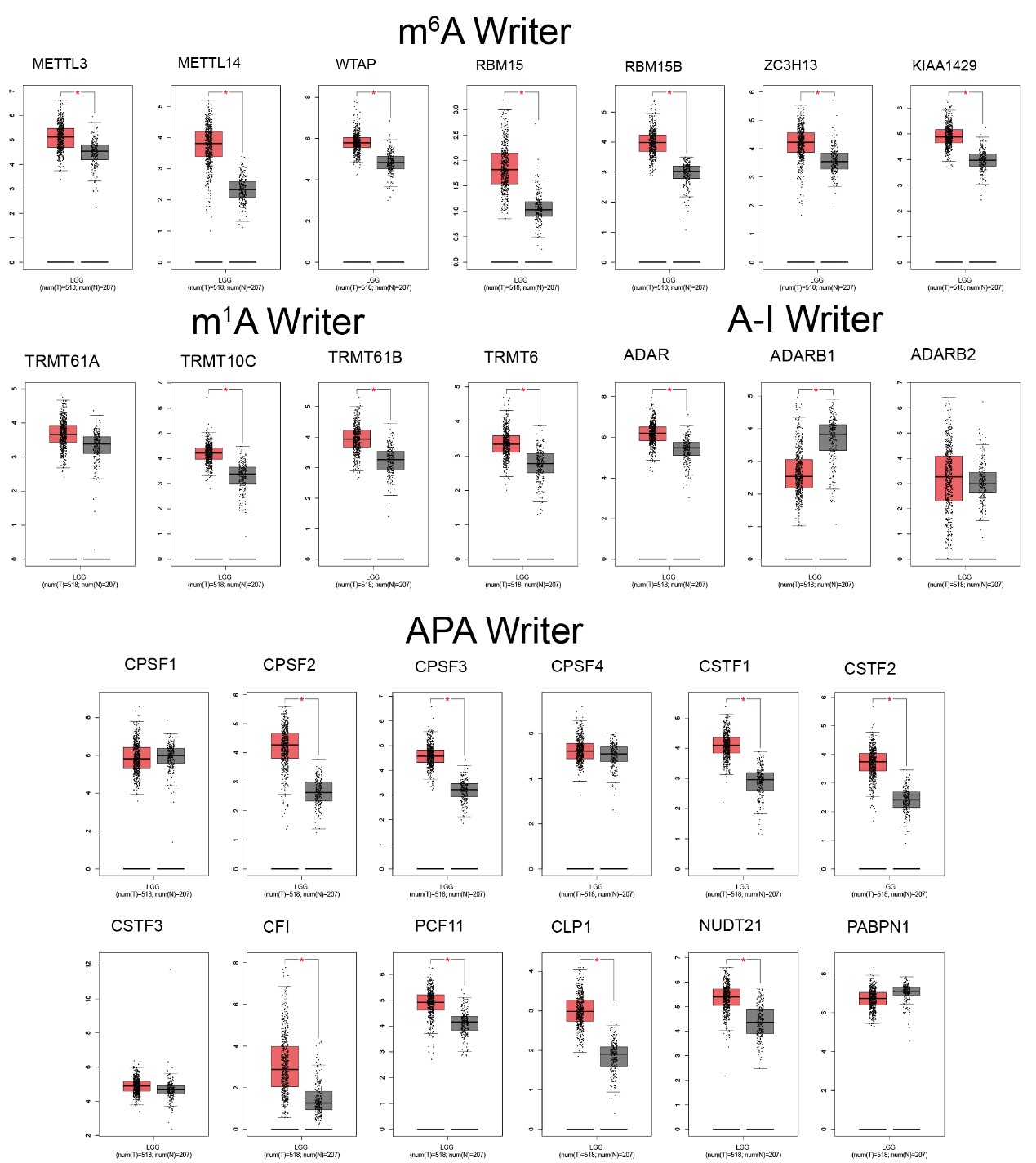
**

**Supplementary Figure 1.** Expression distribution of 26 RNA modification "Writer" enzyme genes in normal tissues and LGG tissues.


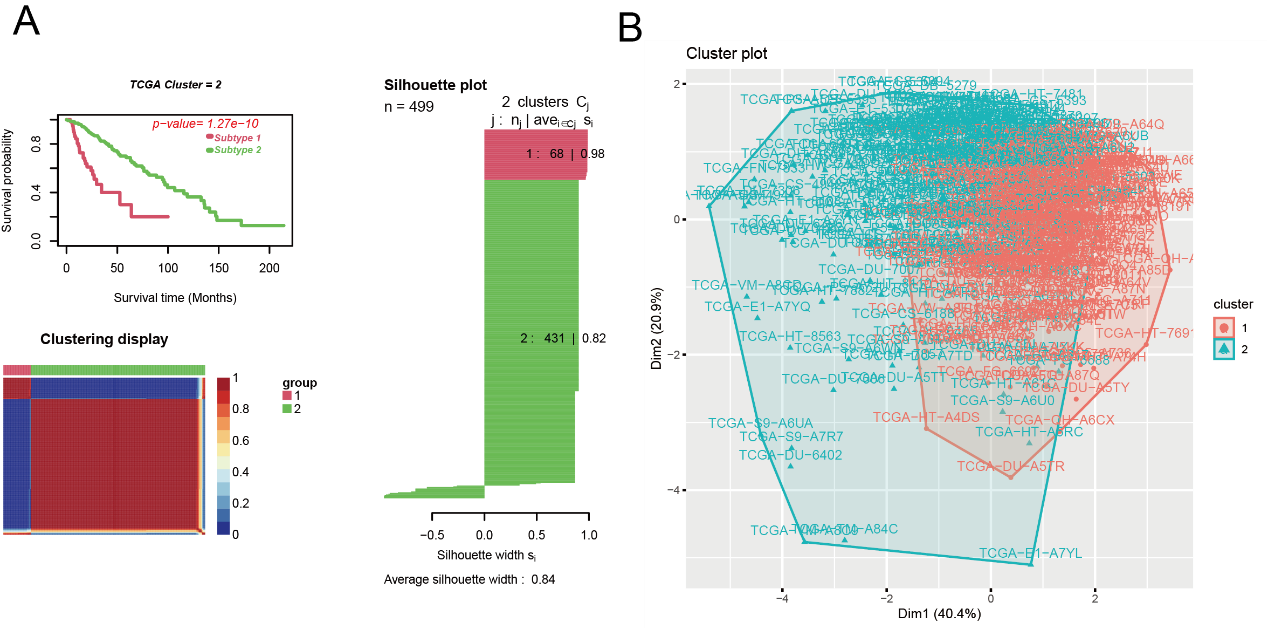


**Supplementary Figure 2.** Identification of RNA modification subtypes in the TCGA-LGG cohort.


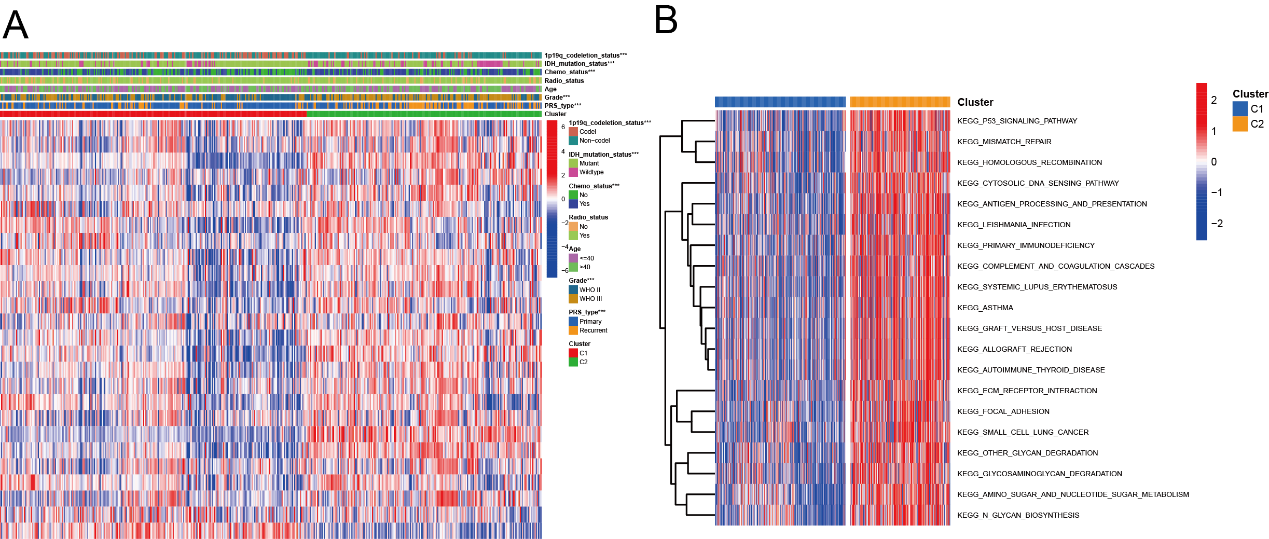


**Supplementary Figure 3.** Differences in clinical characteristics (A) and biological processes (B) between the two RNA modification subtypes.


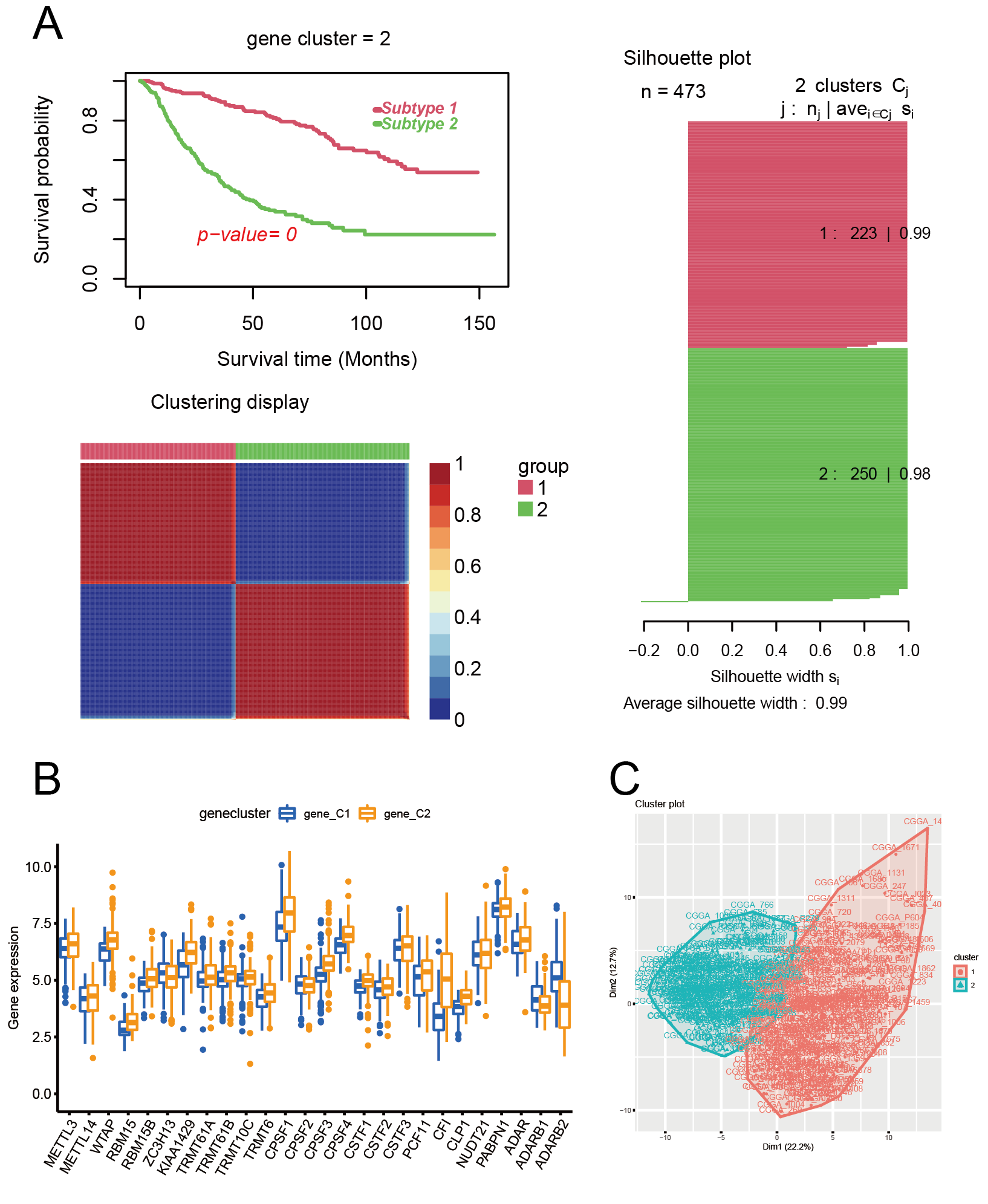


**Supplementary Figure 4.** Gene subtypes identification of characteristic genes based on RNA modification.


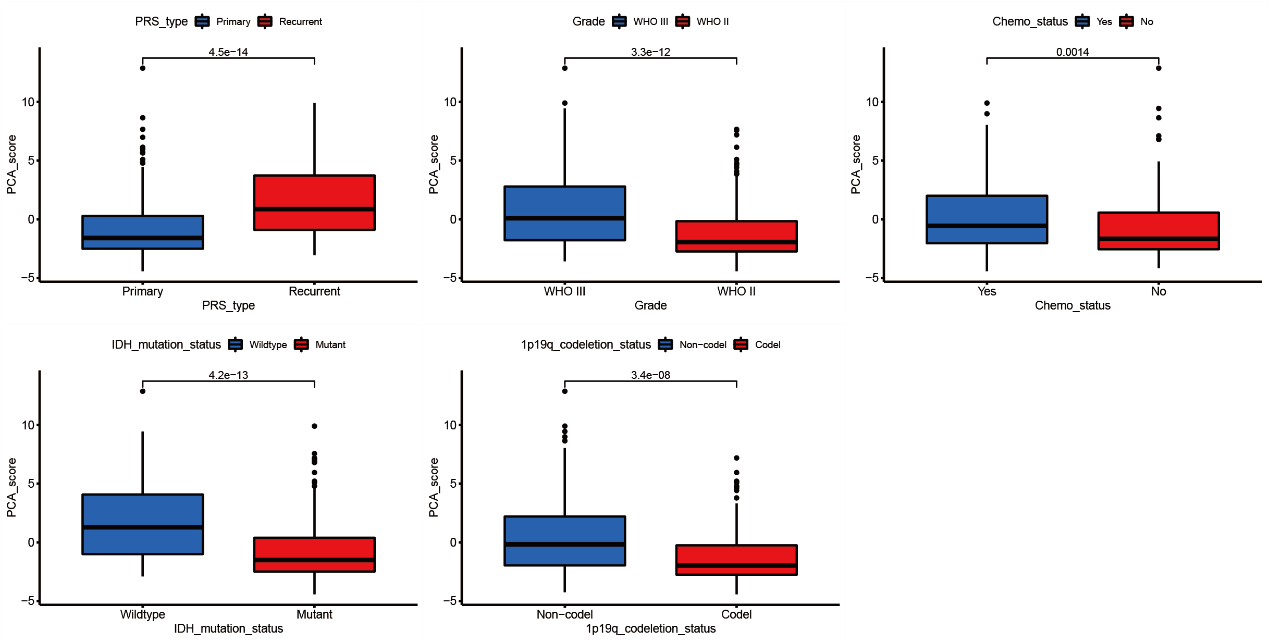


**Supplementary Figure S5.** Differences of PCA_score in different clinical traits.


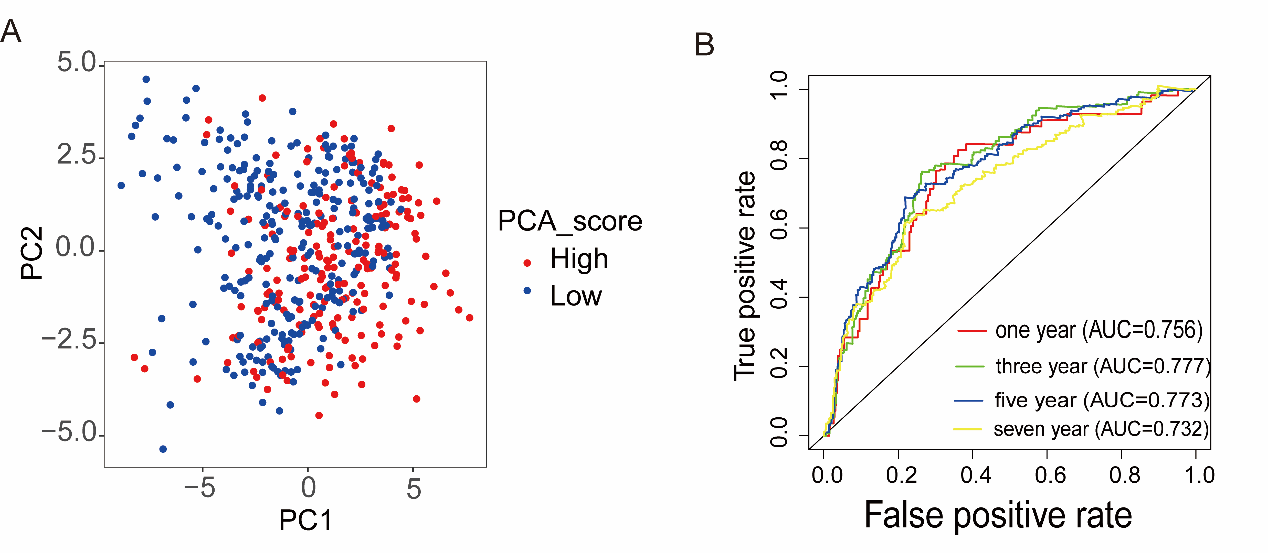


**Supplementary Figure S6.** PCA analysis and ROC curve analysis. (A) PCA analysis based on the prognostic signature. The high- and low-PCA_score patients are represented by red and steel blue dots, respectively. (B) ROC curves to predict the sensitivity and specificity of 1-, 3-, 5-, and 7-year survival according to the PCA_score.


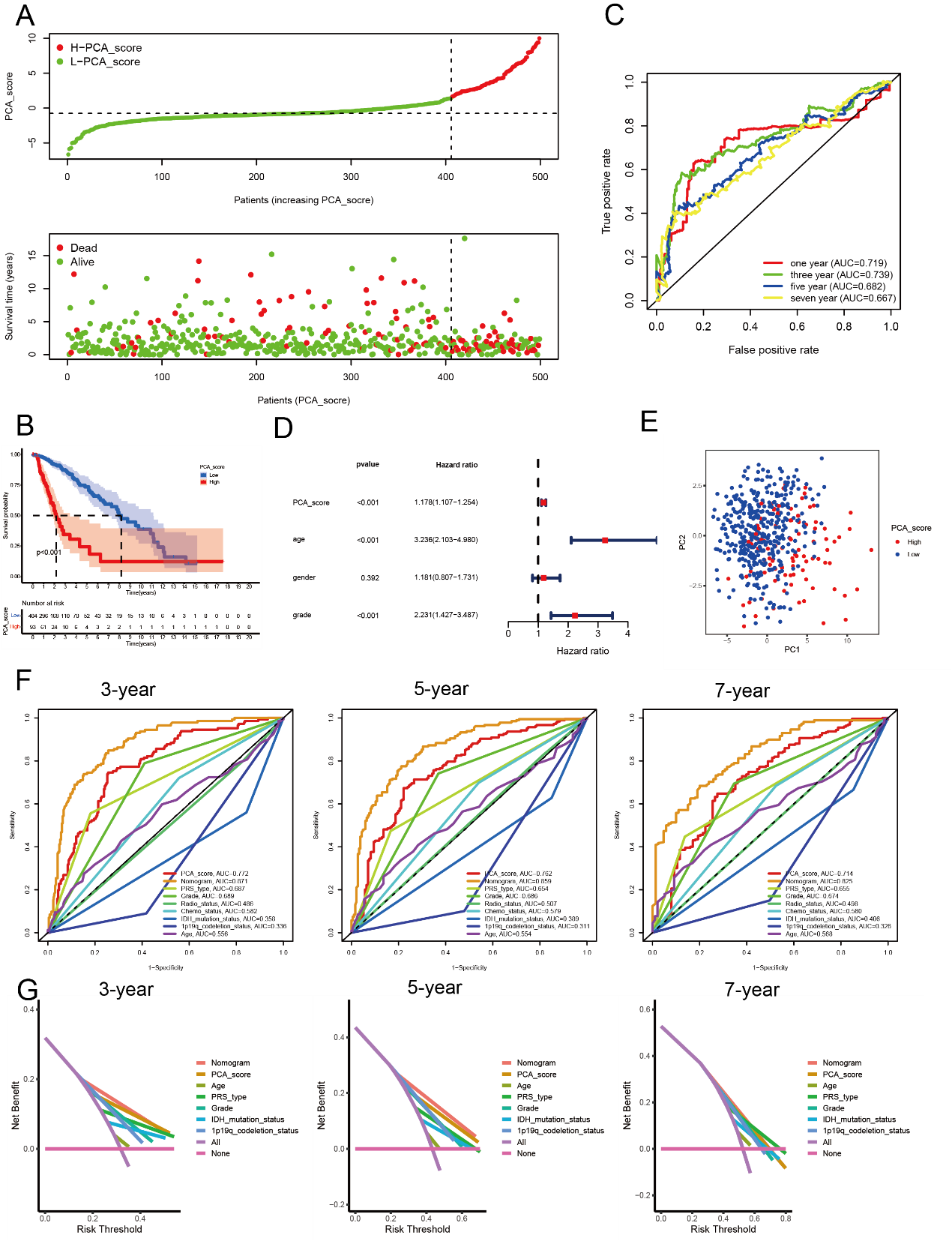


**Supplementary Figure 7.** Validation of the PCA_score prognostic model of the TCGA-LGG cohort.


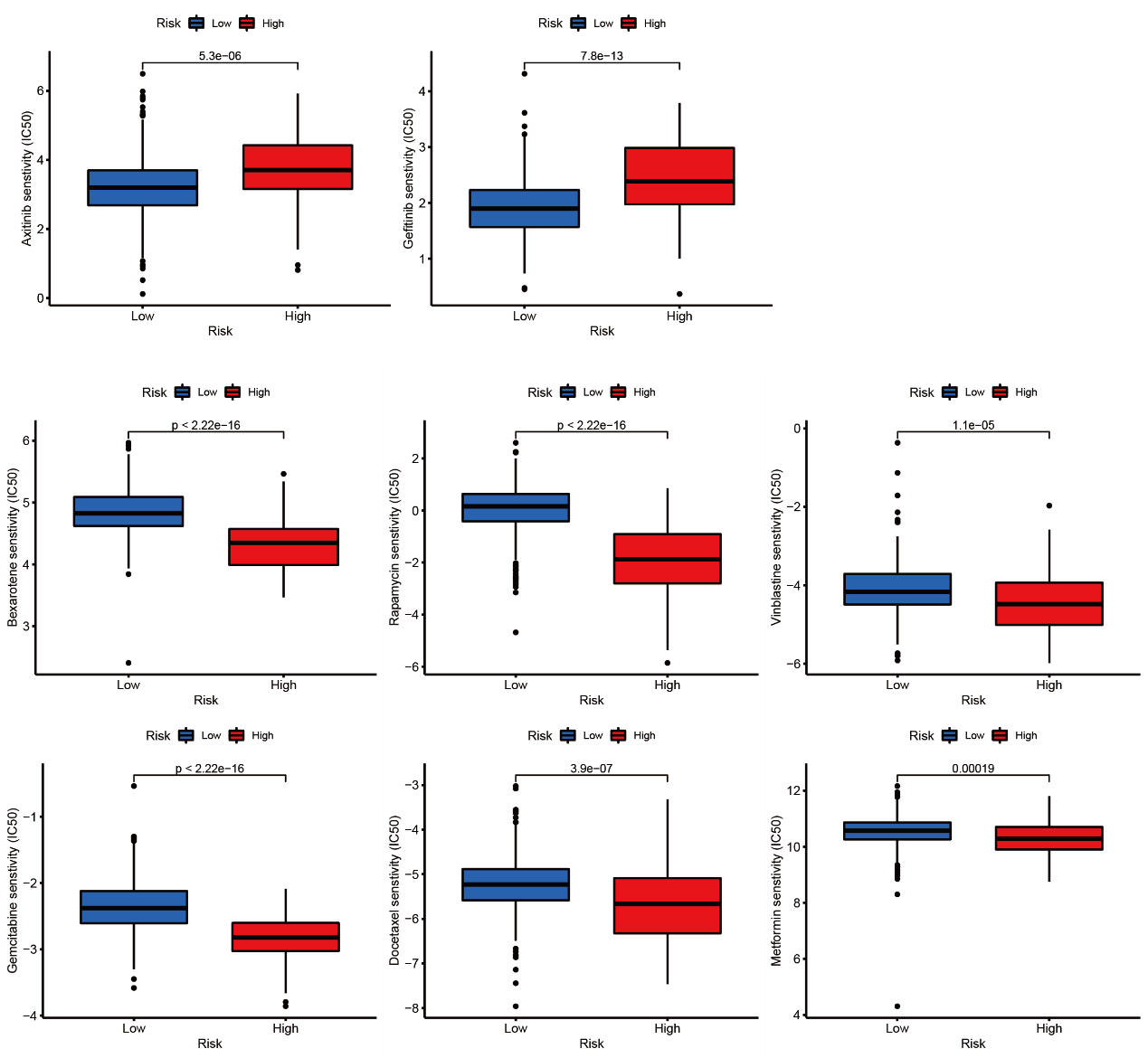


**Supplementary Figure 8.** Correlation between PCA_score and IC50 of common Chemotherapy drugs.


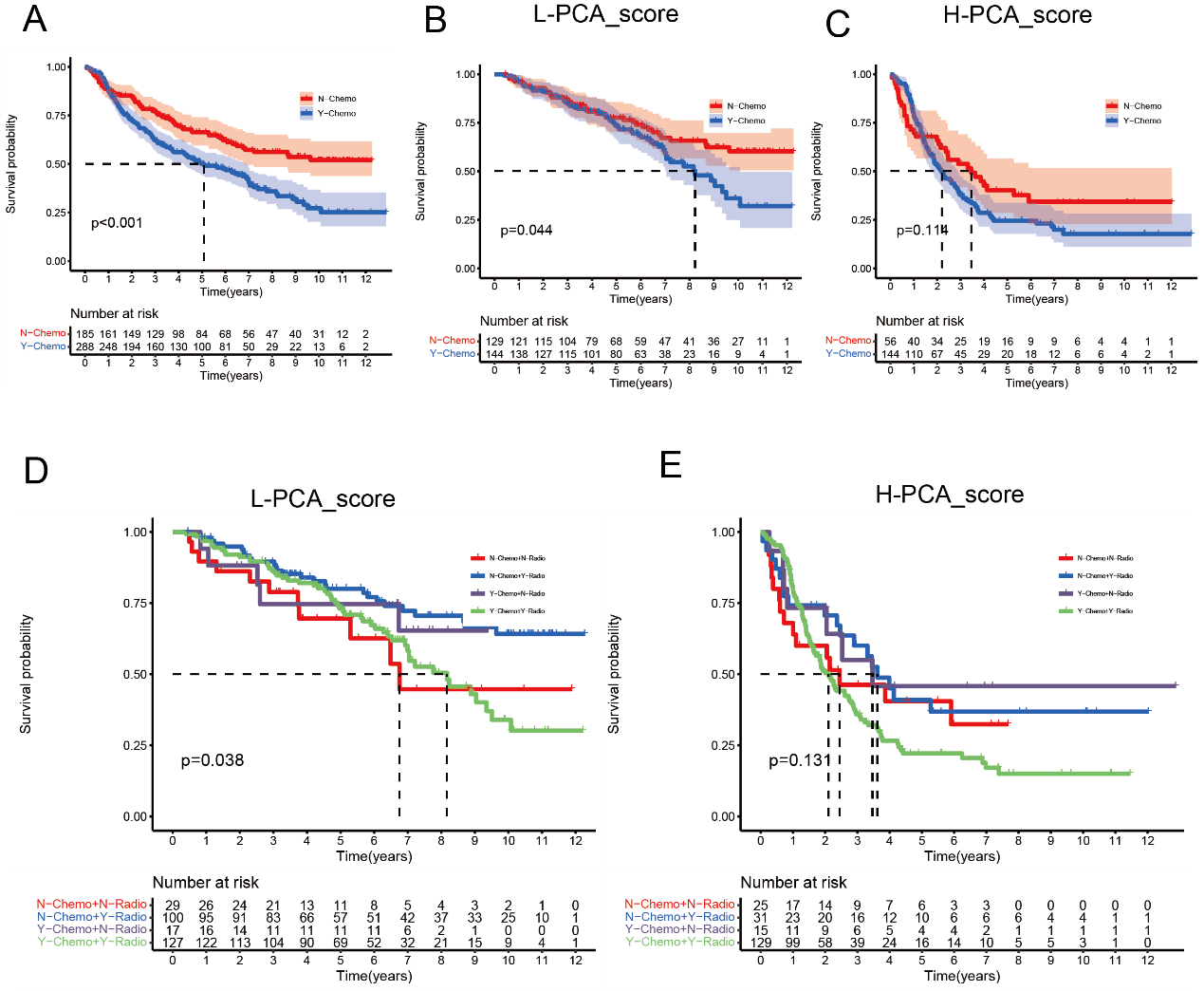


**Supplementary Figure 9.** Kaplan–Meier survival curve of Radio group and non-Chemo group in H-PCA_score.

**Supplementary Figure 9.** the gene feature selection process of the VNLHHO.
