# Supplementary figures and images for: Association of RNA-modification “writer” genes with prognosis and response to immunotherapy in patients with low-grade glioma
Source: PLoS One. 2023 Jan 17;18(1):e0279119. doi: 10.1371/journal.pone.0279119 (PMC9844866; doi:10.1371/journal.pone.0279119)

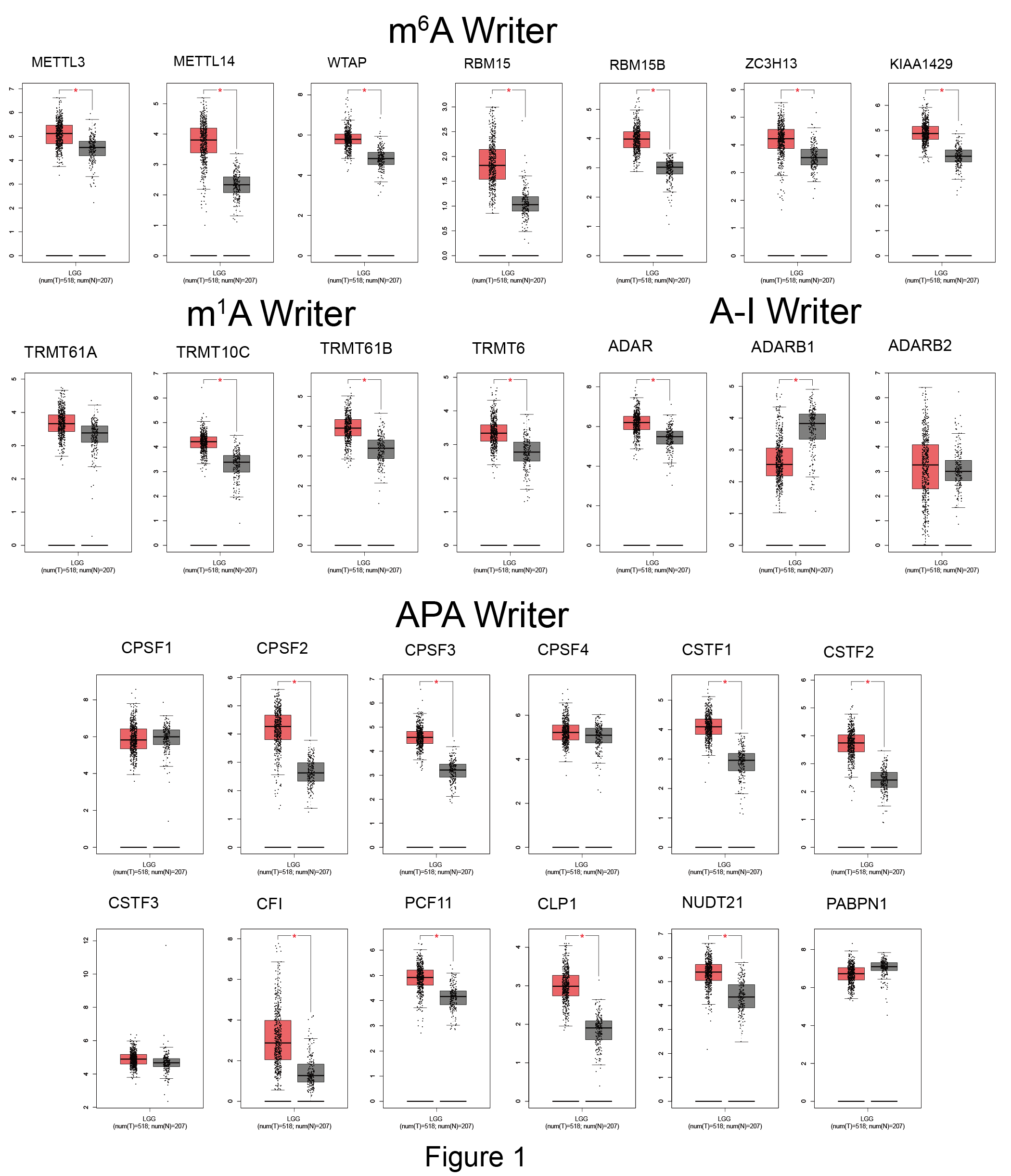

Supplement: S1 Fig — (TIF) [file pone.0279119.s004.tif]

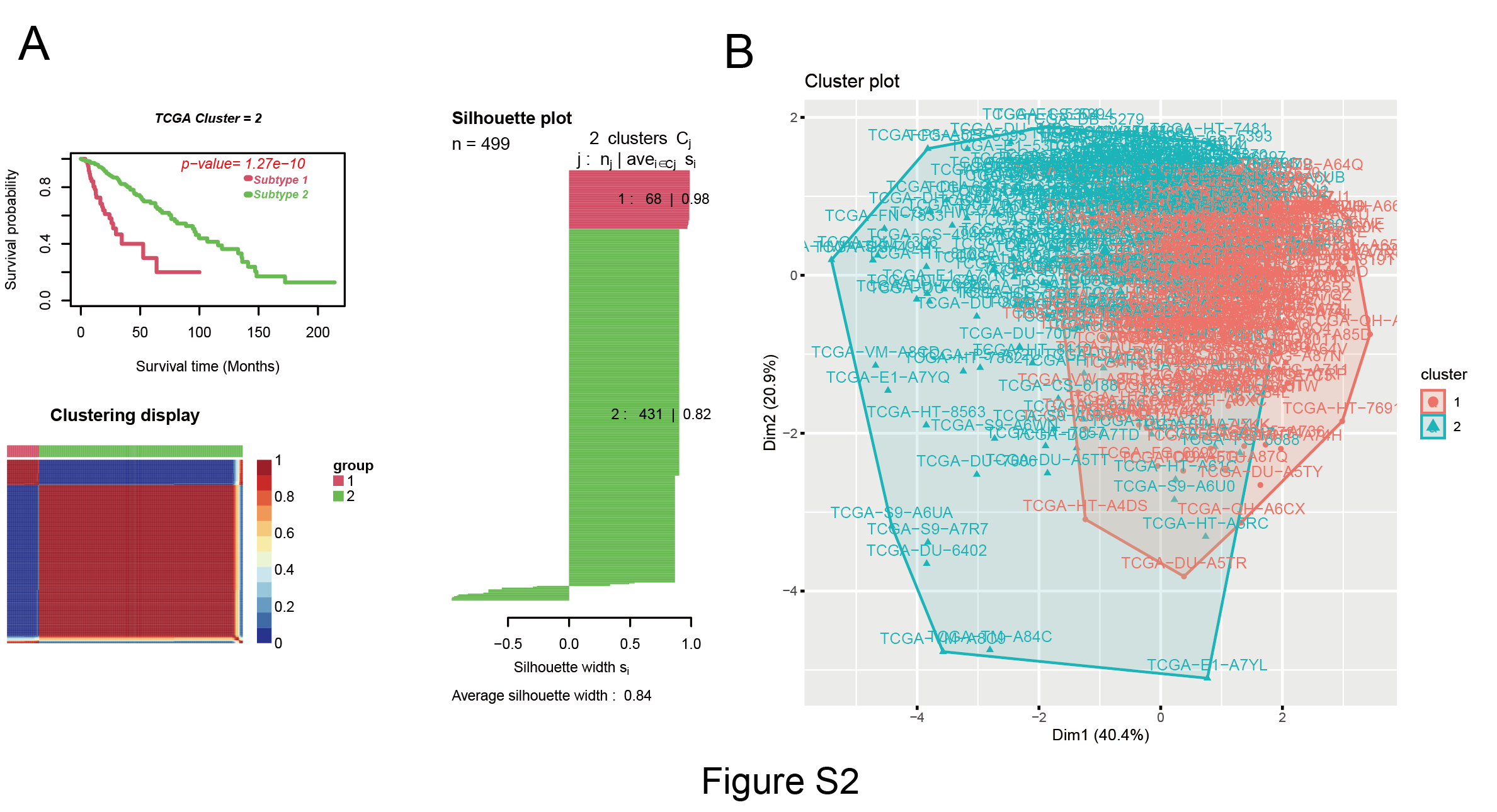

Supplement: S2 Fig — (TIF) [file pone.0279119.s005.tif]

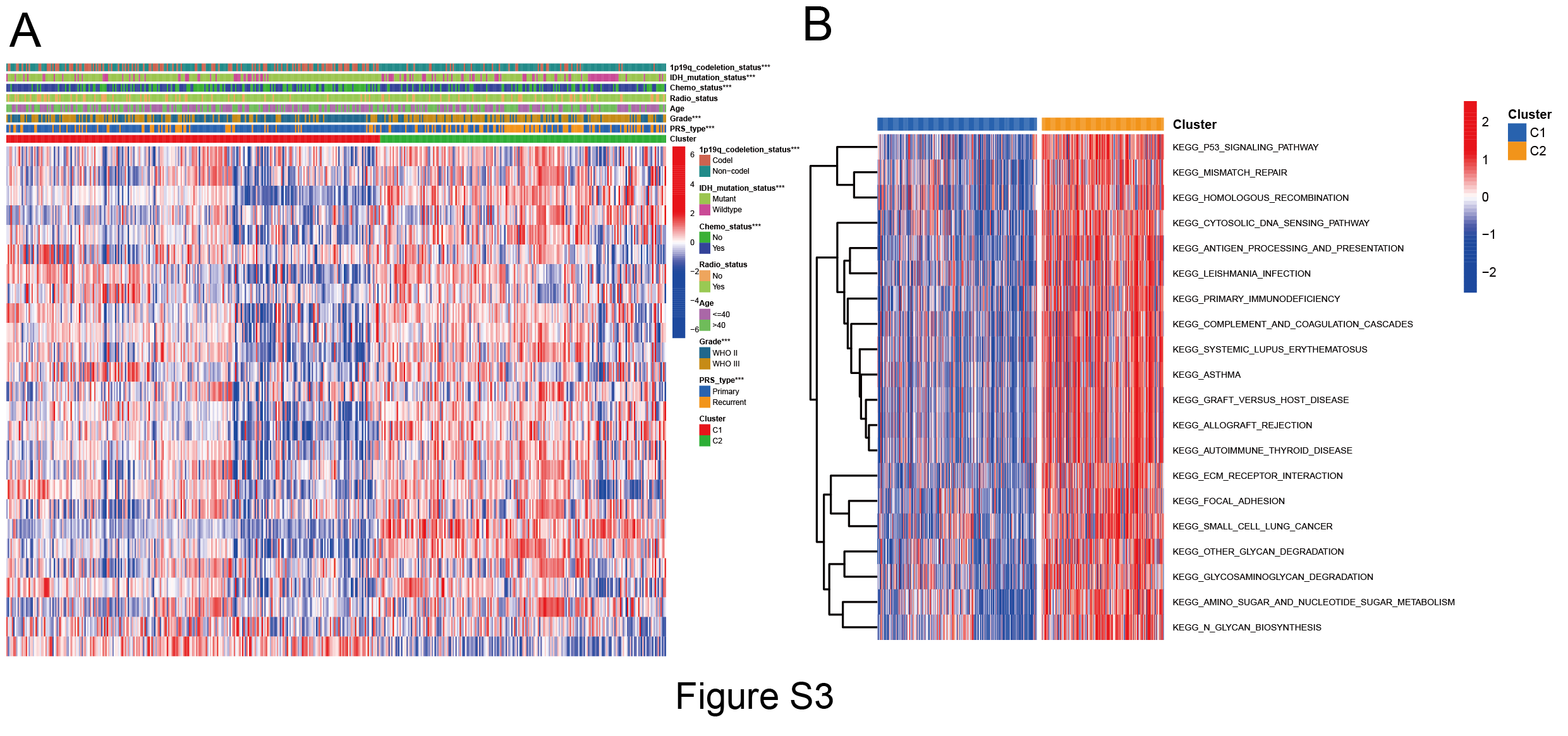

Supplement: S3 Fig — Differences in clinical characteristics (A) and biological processes (B) between the two RNA modification subtypes. (TIF) [file pone.0279119.s006.tif]

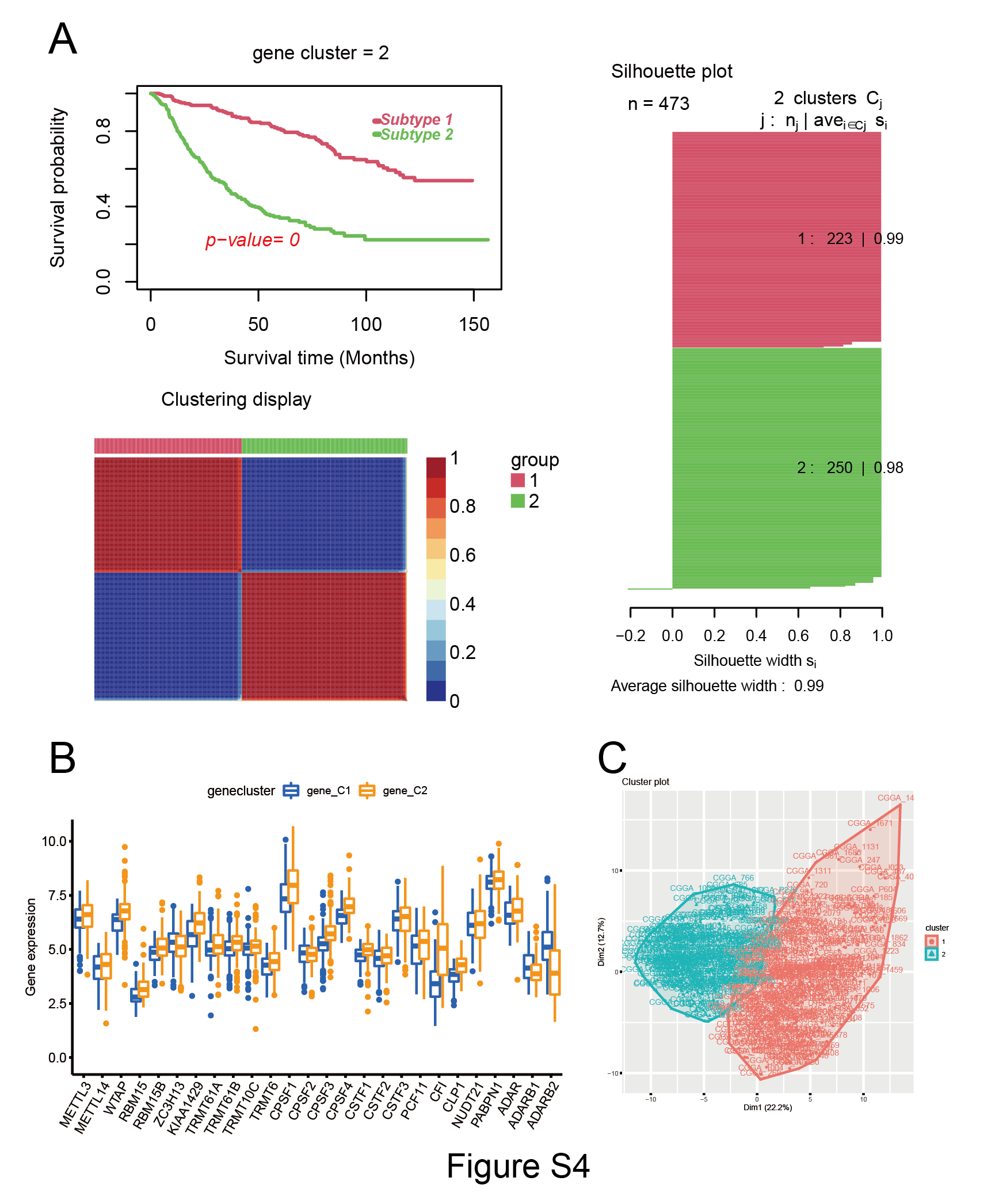

Supplement: S4 Fig — (TIF) [file pone.0279119.s007.tif]

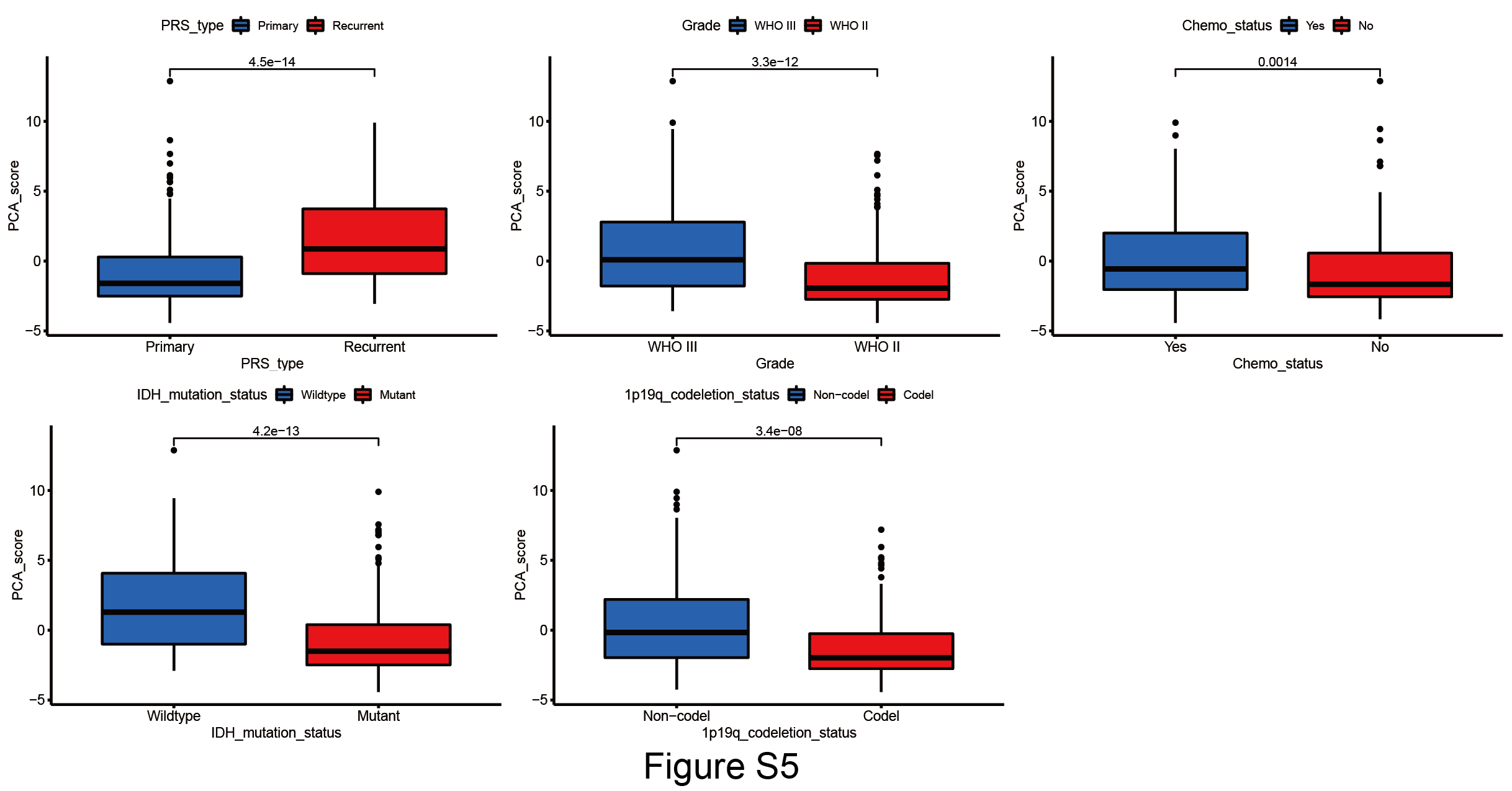

Supplement: S5 Fig — (TIF) [file pone.0279119.s008.tif]

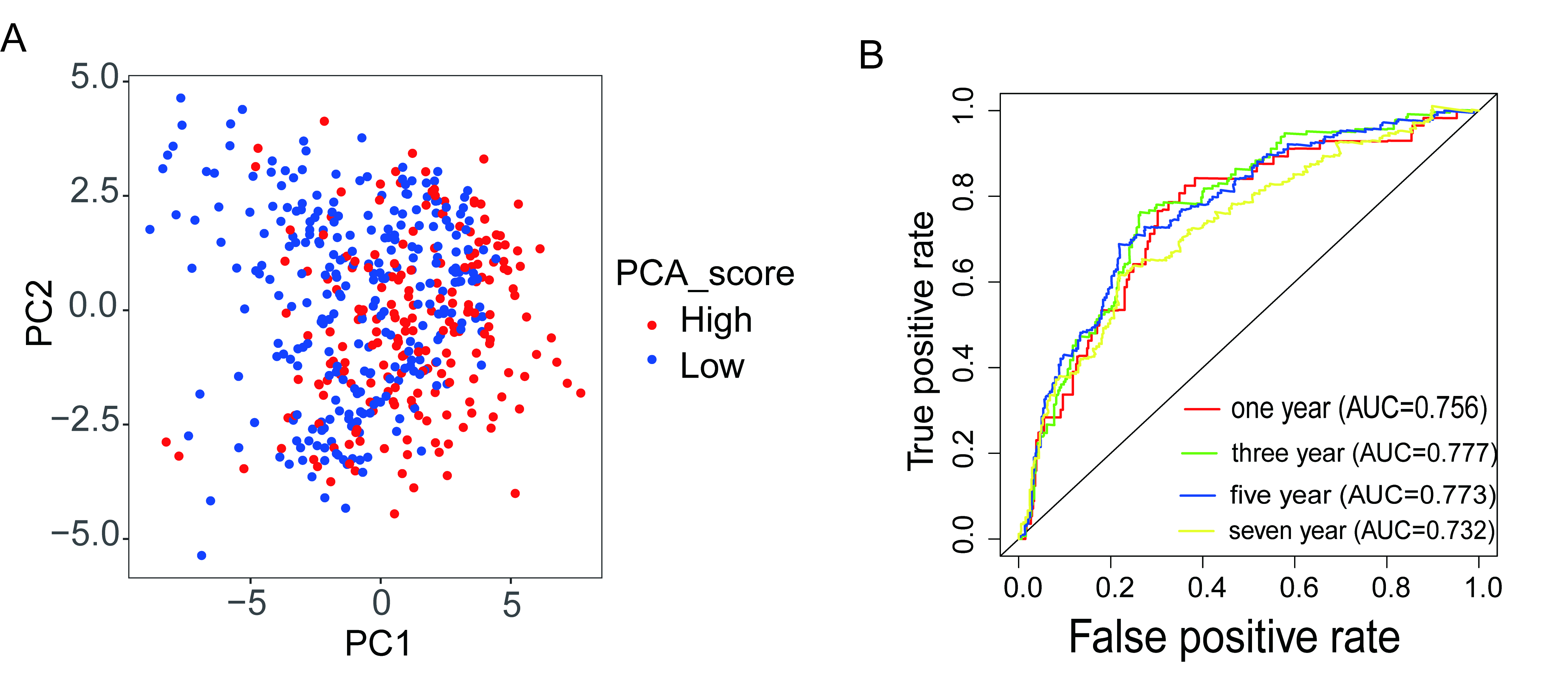

Supplement: S6 Fig — (TIF) [file pone.0279119.s009.tif]

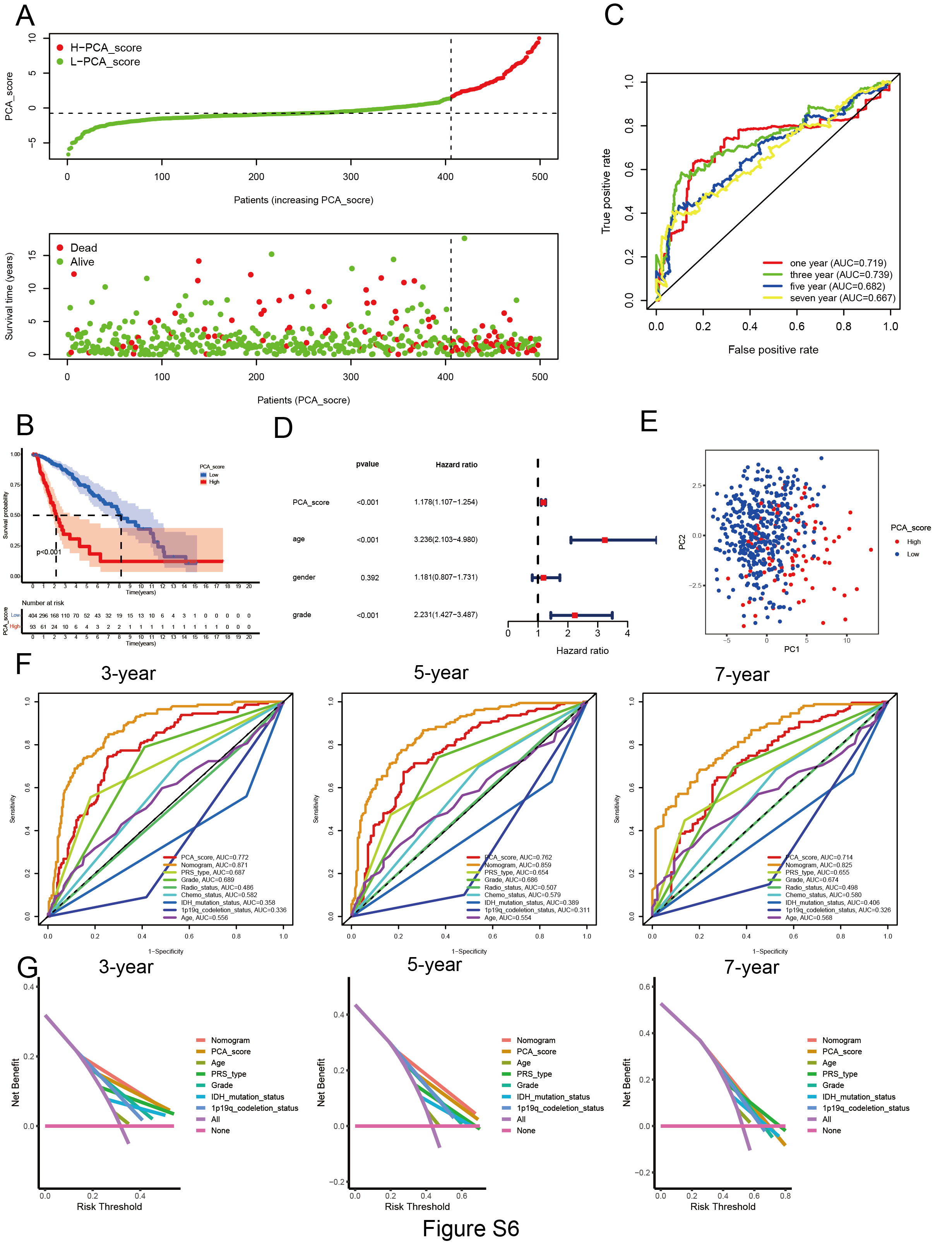

Supplement: S7 Fig — (TIF) [file pone.0279119.s010.tif]

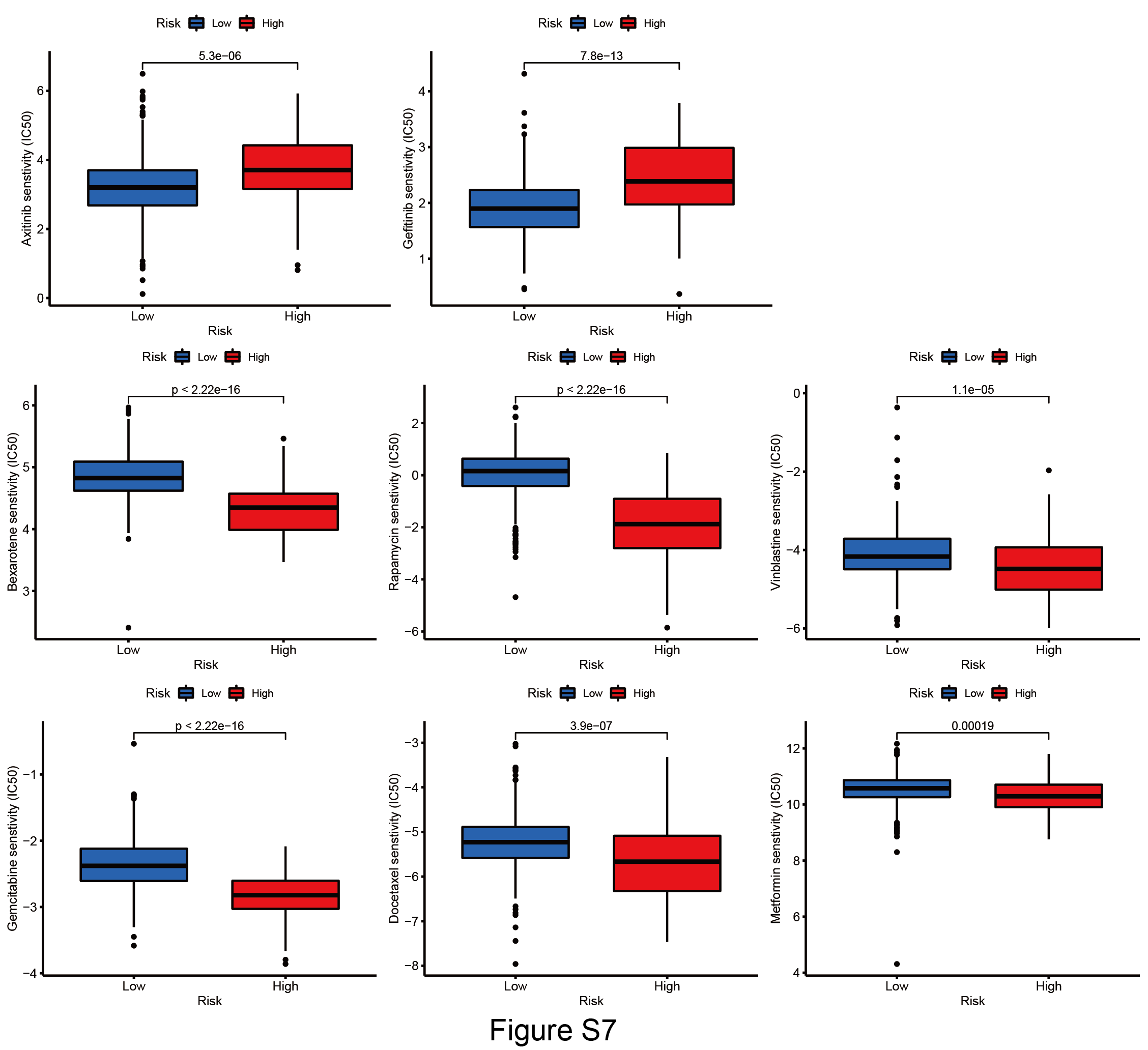

Supplement: S8 Fig — (TIF) [file pone.0279119.s011.tif]

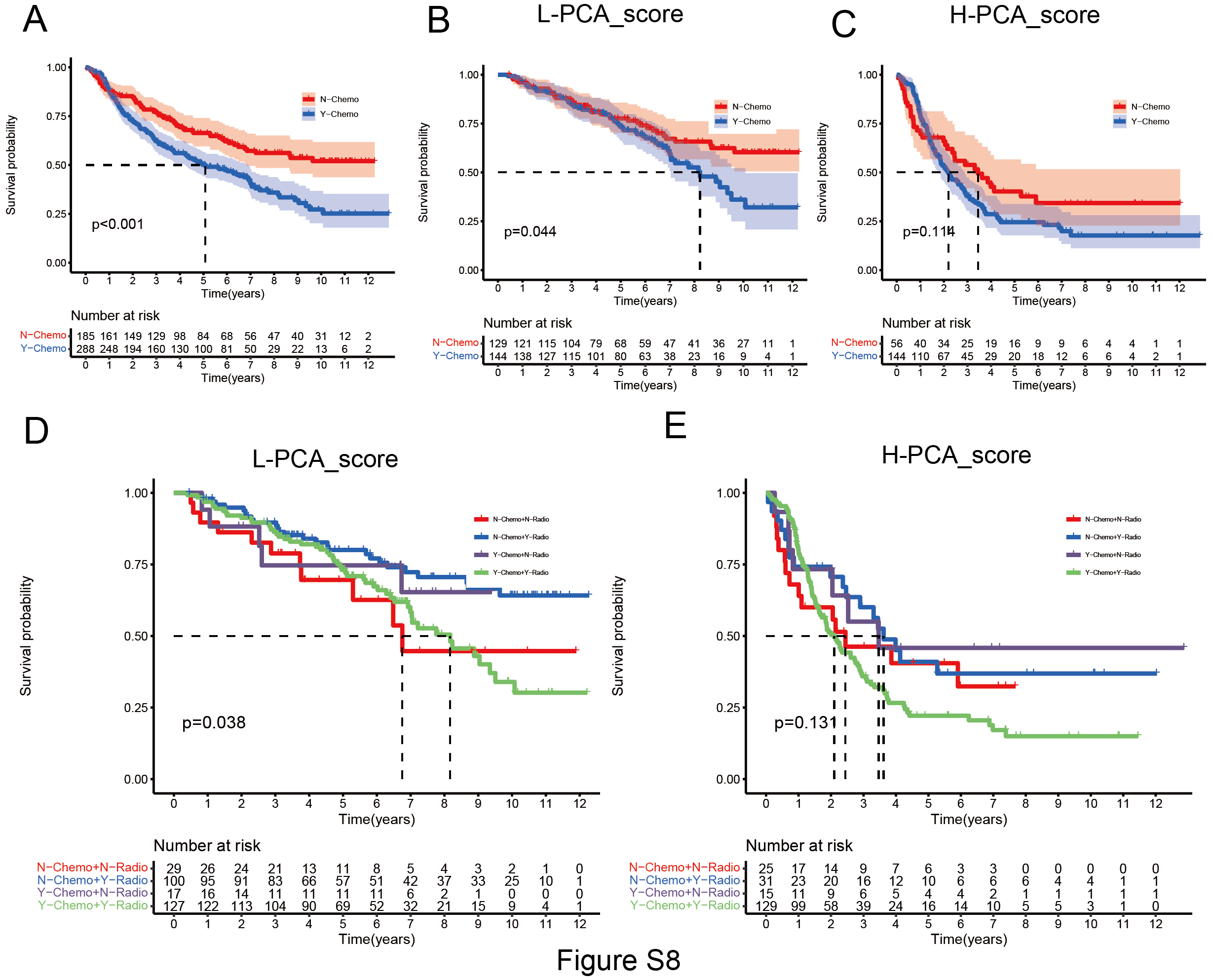

Supplement: S9 Fig — (TIF) [file pone.0279119.s012.tif]
