# Supplementary material for: Association of RNA-modification “writer” genes with prognosis and response to immunotherapy in patients with low-grade glioma
Source: PLoS One. 2023 Jan 17;18(1):e0279119. doi: 10.1371/journal.pone.0279119 (PMC9844866; doi:10.1371/journal.pone.0279119)
Supplement: Attachment — Submitted filename: Response to Reviewers.docx [file pone.0279119.s013.docx]

**Response to Reviewer**

**Reply to Reviewer 1**

**Point 1:** The main concern for this paper from the technical point of view is that the included data from TCGA and CGGA databases are not included in the supplementary materials. Moreover, for reproducibility purposes, readers need to access the R codes used in this research. Many R packages were mentioned in the paper to conduct the analysis, such as "CancerSubtypes, "SVA", "CNMF", "survival", "limma", "cluster profile", "rms", "maftools", “pRRophetic”, "IMvigor210CoreBiologies"," DEseq2", but I was not able to find any of them in the supplementary materials either.

Other than these two issues, the logic behind the analysis and explanation of each one seems reasonable.

**Response :** Thanks for the insightful suggestion. Based on your comments, we have supplemented the corresponding data and codes to make our results more convincing. The code for the key steps used in this paper has been compiled and added to the Supplementary Material. Also, since the data we used came from publicly available databases, they were not uploaded. These data can be found freely at TCGA (<https://portal.gdc.cancer.gov/> ) and CGGA (<http://www.cgga.org.cn/>).

**Reply to Reviewer 2**

**Point 1:** The language of the manuscript is extremely ambiguous and not scientific.

I suggest the authors to restructure and rewrite the abstract clear the message as well as to make the language more scientific, less complex and free of most grammatical and typographical errors.

**Response :** Thank you very much for your criticism. To improve the quality of the manuscript, we have made extensive revisions to the manuscript and marked all revisions in the text. We have rewritten the Abstract section and carefully proofread the manuscript to minimize typographical and grammatical errors.

**Point 2:** A lot of abbreviations have been used but there is no table or figure for the full forms. The abbreviated words have not been described even at the first place.

**Response :** Thanks for your detailed suggestions. We reviewed the full text thoroughly, added a list of abbreviations before the abstract, and described abbreviations in detail when they first appeared.

**Point 3:** The scientific background and rationale of the investigation, and the methodology in the manuscript is satisfactory but needs more description. I would suggest the authors to provide the clear aim, hypothesis and objectives of the study.

**Response :** Thank you very much for your professional advice. We have described our background in detail and provided clear research aim and hypothesis.

**Point 4:** The analysis of the results and the Bioinformatics as well as statistical methods applied in the manuscript are satisfactory and all the data presented in the manuscript is descriptive, and the results are presented in the forms of figures. However, almost 99% of the figures need to be restructured, and the legends should be readable (figure 1B, 1D-nothing is visible, 2A, 2B, 3B, C D, 4A, B, C, D, 5C, D, E, F, G, H, 6 A, B, C, D, E, F). There is repetition of some figures.

**Response :** Thanks a lot. We have reviewed all the Figures in the text, reordered the numbers, and adjusted the legend section to make it readable.

**Point 5:** All the key results should be summarized with reference to study objectives.

**Response :** Thank you very much. We have summarized the main findings with reference to the research objectives and annotated them in the text.

**Point 6:** The interpretation of some of the results is in the light of original results but the limitations of the study have not been discussed. There are several places where references have not been provided.

**Response :** Thanks for your professional review. we have elaborated on the limitations of this study in the Discussion. In addition, corresponding supplements were made where references were lacking. details as follows:

1. We cited references in "Analysis of the correlation between LGG molecular subtypes and the TME" in Methods: Yoshihara K, Shahmoradgoli M, Martínez E, Vegesna R, Kim H, Torres-Garcia W, et al. Inferring tumour purity and stromal and immune cell admixture from expression data. Nature communications. 2013;4:2612. doi: 10.1038/ncomms3612. PubMed PMID: 24113773.

2. We cited references in Methods "Differentially expressed gene (DEG) identification, functional annotation and VNLHHO": Ritchie M, Phipson B, Wu D, Hu Y, Law C, Shi W, et al. limma powers differential expression analyses for RNA-sequencing and microarray studies. Nucleic acids research. 2015;43(7):e47. doi: 10.1093/nar/gkv007. PubMed PMID: 25605792.

3. We cited references in the method "Analysis of the correlation of PCA_score with LGG somatic mutations and drug sensitivity": Geeleher P, Cox N, Huang R. pRRophetic: an R package for prediction of clinical chemotherapeutic response from tumor gene expression levels. PloS one. 2014;9(9):e107468. doi: 10.1371/journal.pone.0107468. PubMed PMID: 25229481.

**Point 7:** There is no experimental validation which can support the findings of the study.

**Response :** This issue is one of the limitations of this study. We will further validate our findings in future work so that the findings are more convincing.

**Point 8:** The most important and major concern is the inclusion of urothelial cancer data in the manuscript. The manuscript is about low-grade glioma so why the authors have provided the methodology (page number 9 and 10) and figures (Figure 8A, B, C, D, E) about urothelial cancer that too without any justification or co-relation.

**Response :** In the present study, we found that PCA_score may be a potential predictor of response to LGG immunotherapy, but no immunotherapy-treated LGG cohort could be used for confirmation. Therefore, we refer to the following articles:

1. Chang P, Chen S, Chang X, Zhu J, Tang Q, Ma L. EXTL3 could serve as a potential biomarker of prognosis and immunotherapy for prostate cancer and its potential mechanisms. European journal of medical research. 2022;27(1):115. doi: 10.1186/s40001-022-00740-w. PubMed PMID: 35818069

2. Peng S, Wang R, Zhou Y, Wei W, Zhong G, Huang X, et al. Insight of a Metabolic Prognostic Model to Identify Tumor Environment and Drug Vulnerability for Lung Adenocarcinoma. Frontiers in immunology. 2022;13:872910. doi: 10.3389/fimmu.2022.872910. PubMed PMID: 35812404

3. Song P, Li W, Guo L, Ying J, Gao S, He J. Identification and Validation of a Novel Signature Based on NK Cell Marker Genes to Predict Prognosis and Immunotherapy Response in Lung Adenocarcinoma by Integrated Analysis of Single-Cell and Bulk RNA-Sequencing. Frontiers in immunology. 2022;13:850745. doi: 10.3389/fimmu.2022.850745. PubMed PMID: 35757748.

4. Zhang H, Deng S, Pi Y, Guo J, Xi H, Shi X, et al. Identification and Validation in a Novel Quantification System of Ferroptosis Patterns for the Prediction of Prognosis and Immunotherapy Response in Left- and Right-Sided Colon Cancer. Frontiers in immunology. 2022;13:855849. doi: 10.3389/fimmu.2022.855849. PubMed PMID: 35444656.

These authors identified potential predictors of immunotherapy response in different cancers, and then used the IMvigor210 cohort to validate the predictors for immunotherapy response.

**Point 9:** I would recommend the authors to restructure and re-write the article and remove all the data from other studies. After the inclusion of all the correction, the manuscript should be sent to an expert of English language.

**Response :** According to your suggestion, we have rewritten this manuscript. For example, we added a list of abbreviations and rewrote the summary section. We rewrote the introduction to describe the background in more depth and clarify the purpose of this study. We rewrote the discussion part of this manuscript, discussed the limitations of this study, and summarized the results of this study. Finally, we polished the manuscript in a professional English editing company to correct English language, grammar, punctuation and phrasing to improve the clarity and readability of academic papers.
