# Supplementary material for: Association of RNA-modification “writer” genes with prognosis and response to immunotherapy in patients with low-grade glioma
Source: PLoS One. 2023 Jan 17;18(1):e0279119. doi: 10.1371/journal.pone.0279119 (PMC9844866; doi:10.1371/journal.pone.0279119)
Supplement: Attachment — Submitted filename: Remarks PONE-D-22-11089_R1_Manuscript 10-03-22.docx [file pone.0279119.s014.docx]

In their article entitled “ "Association of RNA-modification "writer" genes with prognosis and response to immunotherapy in patients with low-grade glioma," Zhang et al. aimed to present a novel RNA modification-based prognostic model for LGG that would serve as the basis for assessing LGG prognosis and creating more potent therapeutic approaches for the LGG.

This constitutes a large, comprehensive, and broadly rational body of work that is appreciable. However, there are a number of minor concerns that can be resolved to improve the quality of the manuscript, listed below.

1. In the line No. 75 the author stated that “due to disease heterogeneity and drug resistance, the prognosis of LGG has not improved significantly”. Please elaborate on the relationship between drug resistance and the prognosis for LGG.
2. The figures are difficult to see and consequently understand, therefore please make extra effort to improve their quality and clarity.
3. The reasoning behind placing the figure legends in the center of the results section. Please create a separate section for all of the combined figure legends.
4. Please explain how these results relate to the validity of the in-vitro, in-vivo implementations.
5. The authors accidentally added their previously submitted revised version (with track change) (from page 60). When submitting to a reputable publication like Plos one, writers are kindly asked to pay more attention to prevent this sort of neglect.

It is a carefully performed study with interesting results and discussion, I would highly recommend the article for publication after the above minor corrections.
