# Supplementary material for: Association of RNA-modification “writer” genes with prognosis and response to immunotherapy in patients with low-grade glioma
Source: PLoS One. 2023 Jan 17;18(1):e0279119. doi: 10.1371/journal.pone.0279119 (PMC9844866; doi:10.1371/journal.pone.0279119)
Supplement: Attachment — Submitted filename: Response to Reviewer.docx [file pone.0279119.s015.docx]

**Response to Reviewer**

**Point 1:** In the line No. 75 the author stated that “due to disease heterogeneity and drug resistance, the prognosis of LGG has not improved significantly”. Please elaborate on the relationship between drug resistance and the prognosis for LGG.

**Response:** Thank you very much for your professional advice. In the second paragraph of the introduction, we have described relationship between drug resistance and the prognosis for LGG in detail and added a new reference for this issue（Wang J, Cazzato E, Ladewig E, Frattini V, Rosenbloom D, Zairis S, et al. Clonal evolution of glioblastoma under therapy. Nature genetics. 2016;48(7):768-76. doi: 10.1038/ng.3590. PubMed PMID: 27270107.）

**Point 2:** The figures are difficult to see and consequently understand, therefore please make extra effort to improve their quality and clarity.

**Response:** Thank you very much for your professional advice. We have remade Figure 1, Figure 2, Figure 3, Figure 4, Figure 5, and improved their clarity. We have renamed the previous version of Figures 5B and 5D as Figure S6. The previous Figure S6, Figure S7, and Figure S8 were changed to Figure S7, Figure S8, and Figure S9 in turn.

**Point 3:** The reasoning behind placing the figure legends in the center of the results section. Please create a separate section for all of the combined figure legends.

**Response:** That’s ture. We added a separate section for all of the combined figure legends.

**Point 4:** Please explain how these results relate to the validity of the in-vitro, in-vivo implementations.

**Response:** Thanks a lot. In our discussion, we added a description of how to use *in vivo* and *in vitro* experiments to confirm the validity of our research results.

**Point 5:** The authors accidentally added their previously submitted revised version (with track change) (from page 60). When submitting to a reputable publication like Plos one, writers are kindly asked to pay more attention to prevent this sort of neglect.

**Response:** Thanks a lot. We have correctly uploaded the second revised version of our manuscript.
